# In Vitro Antibacterial and Antifungal Potential of Amyrin-Type Triterpenoid Isolated from *Datura metel* Linnaeus

**DOI:** 10.1155/2021/1543574

**Published:** 2021-09-18

**Authors:** Sami Bawazeer, Abdur Rauf

**Affiliations:** ^1^Department of Pharmacognosy, Faculty of Pharmacy, Umm Al-Qura University, Makkah, P.O. Box 42, Saudi Arabia; ^2^Department of Chemistry, University of Swabi, Swabi, KPK, Pakistan

## Abstract

The aim of the present investigation was to evaluate the effect of the combined crude extract, fractions, and compound 1 isolated from the fruits of *Datura metel* against selected microbial (bacteria and fungi) strains. Results of antibacterial screening indicated marked susceptibility of the extract and its fractions against tested bacterial strains. Among the extract and various fractions, the chloroform fraction exhibited a significant effect against different bacterial strains including *Escherichia coli*, *Staphylococcus aureus*, and *Bacillus subtilis* with an inhibitory zone ranging from 18 to 24 mm. Similarly, results of antifungal activity revealed that the chloroform fraction displays a promising effect against various fungal strains. The chloroform fraction was subjected to repeated chromatography analysis, which yielded compound **1** (daturaolone). Daturaolone exhibited potent activity against selected bacterial strains including *Klebsiella pneumoniae*, *B. subtilis*, *S. epidermidis*, and *S. aureus* with an inhibitory zone ranging from 12 to 30 mm. In addition, the extracts and daturaolone exhibited significant sensitivity against *T. longifusus*, *C. albicans*, *A. flavus*, *M. canis*, *F. solani*, and *C. glabrata*. Taken all together, it is concluded that our findings validated the traditional use of *D. metel* to treat various infectious diseases, which is supported by daturaolone.

## 1. Introduction

Medicinal plants are the key source of diverse classes of natural products including primary and secondary metabolites, which are produced in plants. Primary metabolites are used by plants for various life functions such as respiration, cell division, growth, reproduction, and photosynthesis among others. On the other hand, secondary metabolites, also known as phytochemicals, natural products, are chemical constitutes which are isolated from plants and utilized for medicinal purposes. In this respect, various plant extracts and phytochemicals are reported for diverse pharmacological potential such as drugs for sexual dysfunction, antioxidants, laxative, antidiabetics, and anticancer agents [1].

*Datura metel* L belonging to the Solanaceae family is locally known as Dhutura. It is an erect shrub that possesses spreading branches and can reach 1.5 meters in height [2]. *D. metel* possesses broadly ovate, simple alternative, glabrous, shallowly lobed, and dark green leaves. Its flowers are solitary, large, and trumpet-shaped, have a sweet smell, and have a wide range of color (yellow to dark purple), which appeared in the evening as well as morning. *D. metel* can be found in tolerate average soil, but it commonly prefers moist soil or alkaline soil. The plant grows in America and is distributed in tropical and subtropical regions, like India and East Asia.

*D. metel* is used as the traditional Bangladeshi herbal medicine [3, 4]. In the Chinese folkloric system, *D. metel* flowers are recognized as baimantuoluo and used in the treatment of skin inflammation and psoriasis. Similarly, *D. metel* seeds are used for the treatment of bronchitis, ulcer, diabetes, and skin rashes. In addition, seeds of *D. metel* are used in the preparation of tea, which is used as sedative, and dried flowers are smoked as cigarettes [5, 6]. Interestingly, *D. metel* produces valuable active secondary metabolites including alkaloids, steroids, triterpenoids, flavonoids, saponins, and tannins [7]. The pharmacological applications of *D. metel* are due to the bioactive phytochemicals. In this context, the alkaloid scopolamine is an important compound extracted from *D. metel.* Scopolamine has been documented for the treatment of different diseases such as asthma and bronchitis [8]. It is also used to cure fever, insanity, epilepsy, heart disease, diarrhea, and diabetes as well as skin diseases [8, 9]. It is also an important source of withanolides, which are used in the treatment of hallucinogenic potency as well as pain [10, 11]. Furthermore, *D. metel* has been reported to relieve tooth pain [12]. In addition, *D. metel* has several uses in the ayurvedic system; several classes of secondary metabolites from this plant are used to treat skin infection and hair fall [7]. Research findings indicated that various solvent extracts of *D. metel* exhibited documented antibacterial, antifungal, and anti-inflammatory properties [13, 14]. Similarly, atropine extracted from *D. metel* has been used to dilate the pupil, and it helps in the surgery of eyes [15]. Based on the preceding discussion, this work is aimed at exploring *D. metel* phytochemically and pharmacologically. In addition, this work deals with the isolation and purification of daturaolone (**1**), along with its in vitro antibacterial and antifungal activity for the discovery of new antimicrobial drugs.

## 2. Material and Methods

### 2.1. Collection of the Plant

Fruits of *D. metel* were collected from the mountain area of Village Razagram Tehsil Khall, District Dir, KPK, Pakistan. Dr. Muhammad Muhammad Ilyas, a botanist at the Department of Botany, University of Swabi, KPK, Pakistan, identified and authenticated the plant. A voucher specimen BOT(UOS-521) was deposited at the herbarium located at the Botany Department, University of Swabi, Pakistan.

### 2.2. Extraction and Isolation of Daturaolone (**1**)

**Figure 1:**
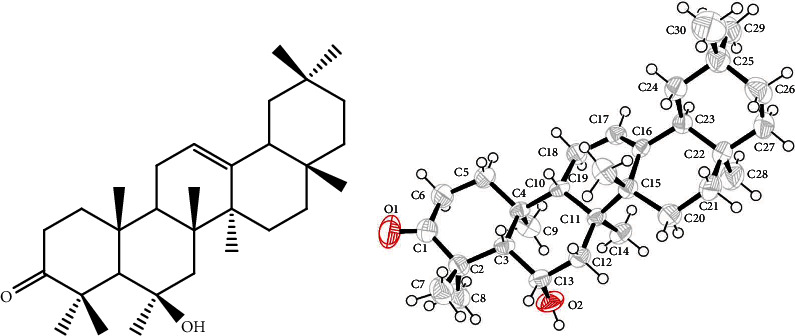
Structure and X-ray crystallographic image of compound 1 isolated from *D. metel*.

The collected fruits (8.00 kg) were washed with water to remove dust particles and then dried in the shade. The dried fruits were ground to fine powder by means of a grinder machine. The powdered material was subjected to cold extraction with commercial grade methanol to afford 349.76 g crude extract. The crude extract was dried at room temperature and was subjected to successive fractionation with various organic solvent and yielded 52.81 g from *n*-hexane, 93.61 g from chloroform, and 45.10 g from ethyl acetate as per our previous reported method [15]. The chloroform fraction was selected for chromatographic analysis, where the column was eluted with mixtures of *n*-hexane and ethyl acetate (0 : 100) with increasing polarity. Two hundred subtractions were collected and compiled based on the TLC profile, which afforded 15 subfractions (SB-1-SB-15). Subfraction SB-7 was subjected to chromatographic analysis to yield white crystals. These crystals were washed with *n*-hexane and regrown in a mixture of *n*-hexane and ethyl acetate, which afforded daturaolone (2.7 g). The chemical structure of daturaolone (Figure 1) was confirmed with the aid of different spectroscopic techniques and by X-ray crystallography [15].

### 2.3. Antimicrobial Screening

#### 2.3.1. Antibacterial Activity

The crude extract, various solvent extracted fractions, and daturaolone were screened for antibacterial activity according to our recently published protocols [12, 16]. Bacterial strains used in this investigation were *Klebsiella pneumoniae* (ATCC 700603), *Escherichia coli* (ATCC25922), *Staphylococcus aureus* (ATCC 25923), *Bacillus subtilis* (ATCC 6633), and *Staphylococcus epidermidis* (ATCC25925) clinical isolates. These bacterial strains were revived from the PNLR Center, Institute of Chemical Sciences, University of Peshawar, KPK, Pakistan. The selected strains were stored in Mueller Hinton agar in the refrigerator at 4°C, prior to culture. The screening of extract/fractions and daturaolone was performed using the agar well diffusion methods according to a standard protocol. In this methodology, Mueller Hinton agar (MHA) was used as the medium. All cultures were taken in triplicate, and the incubation was achieved at standard conditions (37°C, 24–72 h). The prepared cultures (0.6 mL) of the screened organism were placed in a sterile petri dish, and then, 20 mL of sterile molten Mueller Hinton agar (MHA) was added. Then, a hole (6 mm) was bored into the medium using a borer. The standard drug (imipenem), extract, fractions, and daturaolone were introduced to the medium in various concentrations. Incubation was performed for 24 h at 37°C, and the diameters of the inhibitory zone of bacterial growth were recorded in millimeters.

#### 2.3.2. Antifungal Activity

The crude extract, different extract/fractions, and daturaolone were screened for antifungal activity against the fungal strains *Candida albicans*, *Candida glabrata*, *Trichophyton longifusus*, *Aspergillus flavus*, *Fusarium solani*, and *Microsporum canis* clinical isolates using the tube dilution assay according to a published protocol [17]. According to this assay, a stock solution was prepared by dissolving the extract/fractions and isolated compound (**1**) in sterile dimethyl sulfoxide (DMSO). A screw-cap tube was taken, and 6 mL of Sabouraud dextrose was poured in each tube and then put in an autoclave at 20°C for 15 minutes; after that, it was cooled to 15°C. The nonsolidified Sabouraud Dextrose Agar (SDA) was then poisoned with stock solution (66.8 *μ*L) which gives the final concentrations of 400 *μ*g of the extract per mL of SDA. Then, tubes were subjected to solidification in the slanted position at low temperature (25°C). Individually, the tube was then incubated with a piece (4 mm diameter) of inoculums detached from a 7-day old culture of fungi for nonmycelial growth; an agar surface streak was then working. Dimethyl sulfoxide was used as the control while amphotericin and miconazole were used as the reference drug. The inhibition of fungal growth was recorded after 7 days of incubation at 28°C and humidity of 40–50%. The test tubes were scrutinized for the visible growth of fungal strain, and then, the percentage inhibitions were determined.

### 2.4. Statistical Analysis

Assays were conducted in triplicate, and data were subjected to one-way analysis of variance (ANOVA). Results are expressed as the mean ± standard error of the mean (SEM). Statistical analysis was performed using the GraphPad Prism 7 software (GraphPad Software, San Diego, California, USA, https://www.graphpad.com); differences were considered significant at *p* ≤ 0.05.

## 3. Results

### 3.1. Effect of the Extract/Fractions against Bacterial Strain

The results of the extract and various isolated fraction of *Datura metel* Linnaeus fruits against *E. coli*, *K. pneumoniae*, *S. epidermidis*, *S. aureus*, and *B. subtilis* are displayed in Tables 1 and 2. The crude methanolic extract and its subsequent fractions exhibited were found to be sensitive against different Gram-positive and Gram-negative bacterial strains. The chloroform fraction exhibited significant activity against *E. coli*, *S. aureus*, and *B. subtilis*. The methanolic extract showed good activity against *E. coli*, *S. epidermidis*, *S. aureus*, and *B. subtilis* while the ethyl acetate fraction exhibited moderate sensitivity against *Escherichia coli*, *S. epidermidis*, *S. aureus*, and *B. subtilis.* Among the entire fraction, the hexane fraction did not exhibit impressive activity.

### 3.2. Effect of Daturaolone against Bacterial Strains

Results of the antibacterial activity of daturaolone isolated from *D. metel* Linnaeus fruits are shown in Tables 1 and 2. Results revealed that daturaolone exhibits potent activity against *B. subtilis*, significant activity against *E. coli*, and good activity against *S. epidermidis*, *S. aureus*, and *K. pneumoniae.*

### 3.3. Effect of the Extract/Fractions against Fungal Strains

Shown in Table 3 are results of our study of the antifungal activity of the extract and various fractions of *D. metel* Linnaeus. Results indicated that the chloroform fraction exhibits excellent activity against *A. flavus*, *M. canis*, and *F. solani* followed by the methanol extract, which showed good activity against *A. flavus*, *M. canis*, and *F. solani*. On the other hand, the ethyl acetate fraction was sensitive only against *M. canis* while the *n*-hexane was the least active among all fractions.

### 3.4. Antibacterial Effect of Daturaolone

Presented in Table 3 are results of our investigation related to the antibacterial effect of daturaolone isolated from *D. metel*. Results reveal that daturaolone exerts significant activity against *E. coli*, *K. pneumoniae*, *S. epidermis*, *S. aureus*, and *B. subtilis.*

Our findings indicate that the crude extracts/fraction and isolated daturaolone exhibit a broad-spectrum antimicrobial effect against selected pathogens. It has been widely perceived that irrational use of antibiotics could lead to the production of pathogenic bacteria with increasing resistance to different classes of antibiotics. Unfortunately, this has led to progressive loss in the therapeutic efficiency of antibiotics at a rate that varies with complexity of required mutation, the ease of clonal spread, or the rate of horizontal gene transfer [18]. In this context, prevalence of infection due to a multidrug resistance pathogen has shown a dramatic increase around the globe [19]. Several novel antibiotics have been discovered, which are used to combat various types of bacterial infections caused by numerous bacteria, which developed resistance to those antibiotics. In this regard, medicinal plants have been considered a rich source of therapeutic agents for thousands of years [20]. A remarkable number of modern medicine having antimicrobial potency has been prepared from various plants. It has been assumed that plant-based antimicrobial agents may have several possible novel mechanisms of action, which should be important in the prevention/reduction on antimicrobial resistance [21].

In addition, our results demonstrated that the methanol extract and its fractions exhibit promising activity against selected bacteria including *S. epidermidis*, *E.coli*, *S. aureus*, and *B. subtilis*, while the ethyl acetate fraction exhibited moderate sensitivity against *S. epidermidis*, *E. coli*, *S. aureus*, and *B. subtilis.* Similarly, daturaolone exerted potency against tested bacterial strains. The chloroform fraction showed excellent effect against *E. coli*, *S. aureus*, and *B. subtilis* with zones of inhibition ranging from 18 to 24 mm. On the other hand, the *n*-hexane fraction did not exhibit considerable activity. Intestinally, daturaolone exhibited potent activity against *B. subtilis*, significant activity against *E. coli*, and good activity against *S. epidermidis*, *S. aureus*, and *K. pneumoniae* with zone inhibition ranging from 12 to 30 mm.

Similarly, there is a considerable growth in the number of fungus-infected patients throughout the globe; there have been prominent deviations in fungal pathogens. The greatest potential description of these adjustments is the appearance of new fugal pathogens and the growing range with more infections due to fungal pathogens. Possibly, these properties are associated with the increase in the number of patients who are extremely vulnerable to fungal infection. These infections regularly occur in patients with surgery, bone narrow transplant, cancer, and other immunosuppressed patients maximum especially those infected with human immunodeficiency virus (HIV) [21]. In this study, we have screened the extract/fractions and daturaolone for activity against selected fungal strains. Our findings showed that the chloroform fractions exhibited excellent activity against *A. flavus*, *M. canis*, and *F. solani* followed by the methanol extract, which showed good activity against *A. flavus*, *M. canis*, and *F. solani*. On the other hand, the ethyl acetate fraction was sensitive only against *M. canis* while the *n*-hexane was the least active among all fractions. Daturaolone exhibited significant activity against *T. longifusus*, *C. albicans*, *A. flavus*, *M. canis*, *F. solani*, and *C. glabrata* as compared to the standard drug (Table 3). Based on these results, this plant is recommended for further studies that could include isolation of novel natural products as well as derivatization and biotransformation of daturaolone in order to discover novel compounds of clinical applications.

## 4. Conclusions

In summary, findings from this investigation suggest that the fruit crude extracts from *D. metel* and its various fractions exhibit a remarkable and promising antibacterial effect against selected bacterial strains and thus justify the traditional use of the plant for the treatment of numerous diseases. Additionally, the plant extracts exhibited excellent antifungal activity. Furthermore, our findings showed that daturaolone isolated from this medicinal plant exhibited promising antimicrobial activity and could be a lead for the discovery of new antibacterial and antifungal agents. However, more detailed studies are required to establish the safety and efficacy of this plant and to isolate more bioactive compounds.

## Figures and Tables

**Table 1:** Antibacterial effect (zone of inhibition in mm) of the crude extract/fractions and daturaolone isolated from *D. metel* fruits.

Bacteria species	Zone of inhibition in mm						
	Control	Hexane	Chloroform	EtOAC	Methanol	Daturaolone	Imipenem
*E. coli*	0 ± 0.00	0 ± 0.00	18 ± 1.44	12 ± 1.46	16.2 ± 1.23	25.05 ± 0.18	28.24 ± 0.08
*K. pneumoniae*	0 ± 0.00	0 ± 0.00	0 ± 0.00	0 ± 0.00	0 ± 0.00	12.45 ± 00	32.66 ± 0.09
*S. epidermis*	0 ± 0.00	0 ± 0.00	0 ± 0.00	12.98 ± 1.08	18.21 ± 1.90	18.98 ± 0.40	30.76 ± 0.12
*S. aureus*	0 ± 0.00	8.01 ± 1.44	18.65 ± 1.40	10.34 ± 1.44	13.80 ± 1.34	20.32 ± 0.38	32.87 ± 0.18
*B. subtilis*	0 ± 0.00	6.08 ± 1.20	24.29 ± 1.48	8.65 ± 1.49	10.21 ± 1.98	30.08 ± 0.39	34.87 ± 0.30

Data are presented as the mean ± SEM of three sets of individual assays in every column. Standard (imipenem 1 mg), well size 6 mm, and tested sample (2 mg/mL).

**Table 2:** The MIC of the crude extract, various fractions, and daturaolone isolated from *D. metel* fruits.

Bacteria species	MIC (*μ*g/mL)						
	Control	*n*-Hexane	Chloroform	EtOAC	Methanol	Daturaolone	Imipenem
*E. coli*	—	—	143.21 ± 2.00	—	160.23 ± 2.65	2.39 ± 1.00	0.18 ± 0.00
*K. pneumoniae*	—	—	—	—	—	—	0.19 ± 0.04
*S. epidermis*	—	—	—	—	153.21 ± 2.54	28.22 ± 1.88	0.20 ± 0.06
*S. aureus*	—	—	144.32 ± 2.01	—	—	20.11 ± 1.23	0.19 ± 0.02
*B. subtilis*	—	—	12.98 ± 1.64	—	—	0.43 ± 0.34	0.21 ± 0.04

Results are expressed as the mean ± SEM of three sets of individual assays in every column.

**Table 3:** Antifungal effect (zone of inhibition in mm) of the crude extract, different fractions, and daturaolone isolated from *D. metel* fruits.

Fungal species	% zone of inhibition						
	*n*-Hexane	Chloroform	EtOAC	Methanol	Daturaolone	Standard	MIC (*μ*g/mL)
*T. longifusus*	—	—	—	—	—	Miconazole	108.23 ± 1.21
*C. albicans*	—	—	—	—	—	Miconazole	21.56 ± 1.15
*A. flavus*	—	24.23 ± 2.88	—	20.20 ± 2.96	36.77 ± 1.10	Amphotericin B	98.98 ± 0.80
*M. canis*	12.87 ± 2.87	28.98 ± 2.00	16.23 ± 1.87	25.32 ± 1.45	38.98 ± 1.00	Miconazole	73.34 ± 0.64
*F. solani*	—	22.98 ± 1.98	—	16.11 ± 2.88	32.98 ± 1.60	Miconazole	110.12 ± 0.23
*C. glabrata*	—	—	—	—	—	Miconazole	108.23 ± 0.06

Results are expressed as the mean ± SEM of three sets of individual assays in every column.

## Data Availability

The data associated with this paper are given in the main text of this paper. The spectroscopic data of compounds is available from corresponding authors upon request.
